# Sensory Profile and Preliminary Acceptability of Fructose Syrup Produced from Yacon Extract (*Smallanthus sonchifolius*) via Enzymatic Hydrolysis

**DOI:** 10.3390/foods15142553

**Published:** 2026-07-20

**Authors:** Augusto Castillo, Jorge Domínguez, Any Córdova-Chang, Abel José Rodríguez-Yparraguirre

**Affiliations:** Department of Agroindustry and Agronomy, Faculty of Engineering, Universidad Nacional del Santa, Nuevo Chimbote 02712, Ancash, Peru; jdominguez@uns.edu.pe (J.D.); acordova@uns.edu.pe (A.C.-C.); arodriguez@uns.edu.pe (A.J.R.-Y.)

**Keywords:** *Smallanthus sonchifolius*, fructose-rich sweeteners, sensory evaluation, trained panel analysis, natural food ingredients, functional sweeteners

## Abstract

This study reports the psychometric-multivariate characterization of the sensory space of a *Smallanthus sonchifolius* fructose syrup obtained via enzymatic hydrolysis, addressing the need for robust evaluation of novel functional sweeteners. A 12-member selected analytical panel (selected from 38 candidates; 31.6% retention) evaluated the matrix using a 16-item, four-dimensional psychometric instrument. Content validity was pre-established by expert consensus (global Aiken’s *V* = 0.824, 95% CI [0.798, 0.852]), and panel reliability was confirmed through Friedman’s test across three replicates (*p* > 0.05 for D2, D3, D4). Results from Principal Component Analysis (PCA) explained 90.17% of the total variance, identifying two primary axes: somatic-emotional integration (PC1: 64.15%) and contextual-cognitive differentiation (PC2: 26.02%). Hierarchical Cluster Analysis (HCA) segmented three distinct perceptual archetypes within the panel (*k* = 3), with the “Temporally Driven” segment (C3) emerging as the dominant profile (50%). The findings demonstrate that yacon syrup perception is characterized by statistical score homogeneity across oral body-mapping and experiential familiarity dimensions. This psychometric approach provides a high-fidelity map of the sensory experience, offering a reproducible baseline for the quality control and standardization of functional yacon-derived products.

## 1. Introduction

Global demand for reduced-sugar food has driven the evaluation of inulin-accumulating tubers as botanical sources of fructose-based sweeteners [1,2]. Root and tuber matrices with elevated polysaccharide fractions—particularly β (2 → 1)-fructans—offer technological and sensory advantages over refined sucrose and synthetic additives [3,4,5]. Fructose-based sweeteners derived from botanical sources exhibit a sweetening capacity approximately 1.2–1.7× that of sucrose on an equimolar basis, combined with a lower glycemic index, conferring commercial relevance for reformulation in functional food categories [6,7].

Yacon (*Smallanthus sonchifolius*)—a tuberous plant native to the Andean highlands of South America and now cultivated across Europe (Italy, Czech Republic, Portugal), Oceania (New Zealand), South-East Asia (Indonesia), and North America [8,9,10]—accumulates fructooligosaccharides (FOS) and fructan-derived compounds constituting approximately 80% of its carbohydrate fraction (fresh root composition: ~70% water; ~20% total carbohydrates, of which ~80% are inulin-type FOS) [1,11]. These FOS fractions—chiefly kestose (GF_2_), nystose (GF_3_), and fructosylnystose (GF_4_)—confer both prebiotic functionality and a characteristic sweetness profile that distinguishes yacon from sucrose-based systems [3,6,12,13].

Enzymatic hydrolysis via inulinase (EC 3.2.1.7) converts the native FOS fraction to free fructose and shorter-chain oligomers, yielding syrups with concentrated sugar profiles (65–70% total carbohydrates; ~25% water) [14,15]. Process control of hydrolysis duration and enzyme loading determines the residual degree-of-polymerization (DP) distribution, which influences both the sweetening capacity (mediated by free fructose) and the rheological properties of the resulting syrup (mediated by DP ≥ 3 species) [16,17,18]. Ultrasound- and microwave-assisted extraction protocols have been explored to improve the yield and compositional consistency of yacon-derived extracts prior to enzymatic processing [19,20].

Despite compositional advances, the literature on sensory characterization of enzymatically processed yacon sweeteners remains sparse. Most published studies address the anti-inflammatory, antioxidant [21,22], and metabolic [17,23] effects of yacon preparations, or evaluate FOS-enriched syrups in food applications—beverages [6,19], snacks [24], prebiotic coatings [4]—without sensory evaluation of the syrup matrix itself. The few studies addressing sensory aspects of yacon products employ rapid characterization methods (Flash Profile, ComDim) applied to fermented juices or flour-based matrices [25,26], not to enzymatically hydrolyzed fructose syrups. Trained-panel descriptive analysis under controlled sensory evaluation conditions has not been applied to this product category.

To address these methodological and characterization gaps, this study aims to systematically establish the multidimensional sensory profile and preliminary acceptability of a novel fructose syrup produced from yacon extract via targeted enzymatic hydrolysis. We hypothesize that: (i) the enzymatic conversion of yacon fructooligosaccharides into free fructose generates a stable sensory space characterized by distinct, repeatable descriptive dimensions across panel evaluations, and (ii) despite the exploratory nature of the single-batch formulation, the resulting syrup exhibits structural sensory attributes that positively influence initial panel evaluation response. By mapping these dimensions through a validated psychometric framework and multivariate chemometrics, this research seeks to provide a reproducible baseline for the sensory standardization of functional yacon-derived sweeteners.

## 2. Materials and Methods

### 2.1. Raw Material and Fructose Syrup Sample

The choice of a fructose-rich syrup derived from *Smallanthus sonchifolius* as the target matrix was based on its unique technological and functional properties. Unlike conventional sucrose systems, yacon syrup features a highly complex sensory signature characterized by an intricate balance of early sweetness onset (driven by free fructose) and persistent mouthcoating texturization (driven by its fructooligosaccharide fraction), making it an ideal, challenging model for testing multidimensional sensory instruments. A single, tightly controlled production batch was deliberately selected for this study to isolate the psychometric performance of the newly developed evaluation framework from external compositional noise. By keeping the matrix chemical profile strictly constant, the study minimized raw material batch-to-batch variability, allowing for an unconfounded assessment of the panel’s internal consistency, scale calibration, and multivariate perceptual segmentation.

The fructose syrup characterized in this study was produced from a *Smallanthus sonchifolius* extract by inulinase-catalyzed hydrolysis using *Kluyveromyces marxianus* NRRL Y-7571. Tuberous roots (fresh mass: 250–350 g per unit; uniform maturity; no visible surface damage) were sourced from smallholder farms in the Ancash highlands, Peru (~3200 m a.s.l.) and processed within 24 h of harvest. All roots were obtained from the same harvest batch. Following mechanical washing and abrasive peeling, the root parenchyma was homogenized and filtered through a 0.5 mm mesh to obtain a clarified aqueous extract. β-D-Fructan fructanohydrolase (EC 3.2.1.7) activity of 13.11 UI/mL was applied at an enzyme loading of 1028.64 IU in a reaction volume of 800 mL containing 53% (*v*/*v*) yacon extract at 10.83 °Brix. These hydrolysis conditions were selected based on preliminary laboratory-scale experiments conducted prior to the present study, in which enzyme loading and reaction conditions were evaluated to maximize fructose release while maintaining adequate physicochemical stability of the syrup. The selected parameters corresponded to the conditions that produced the most suitable syrup characteristics for subsequent sensory evaluation.

The enzymatic reaction was conducted in custom-built, glass stirred-tank mini-bioreactor (designed and assembled at the Universidad Nacional del Santa, Nuevo Chimbote, Peru) at a controlled temperature at 50 °C for 4 h under constant agitation at 180 rpm, with the initial substrate pH adjusted to 5.0. The resulting syrup presented the following compositional parameters: 15.3 °Brix (refractometric) and final pH 4.7 ± 0.1. The detailed chemical profile of the matrix was determined using standardized analytical assays: reducing sugars were quantified via the 3,5-dinitrosalicylic acid (DNS) spectrophotometric method, and free glucose was measured using a commercial enzymatic colorimetric kit (GOD-POD, Glucose Oxidase–Peroxidase, MonlabTest, Barcelona, Spain; the total fructose concentration was subsequently calculated by mathematical difference. Based on these protocols, the syrup’s composition was established at 46.25 g/L of reducing sugars and 35.22 g/L of free fructose. To minimize biological variability, roots with similar size, maturity, and absence of physical deterioration were selected and subjected to identical storage and processing conditions prior to enzymatic hydrolysis. Syrup aliquots were dispensed into sterile borosilicate vials (25 mL), sealed, and stored at 4 °C ± 0.5 °C until sensory evaluation (maximum storage interval: 72 h).

### 2.2. Psychometric Design and Content Validity of the Instrument

A multidimensional sensory instrument was developed to assess different components of product perception commonly considered in sensory evaluation, including affective responses elicited by sensory stimuli, temporal changes in perception during consumption, oral sensations associated with texture and mouthfeel, and the overall contextual appraisal of the product experience. Based on these conceptual dimensions, the instrument was structured into four analytical perceptual domains: (D1) Neuro-emotional Resonance referring to the affective and hedonic responses associated with product consumption; (D2) Sensory Trajectory, describing the temporal evolution of sensory perceptions during tasting; (D3) Oral Body-Mapping, representing the localization and intensity of oral sensations related to texture, viscosity, and mouthfeel; and (D4) Experiential Context, encompassing the overall subjective evaluation and contextual interpretation of the consumption experience.

**Figure 1:**
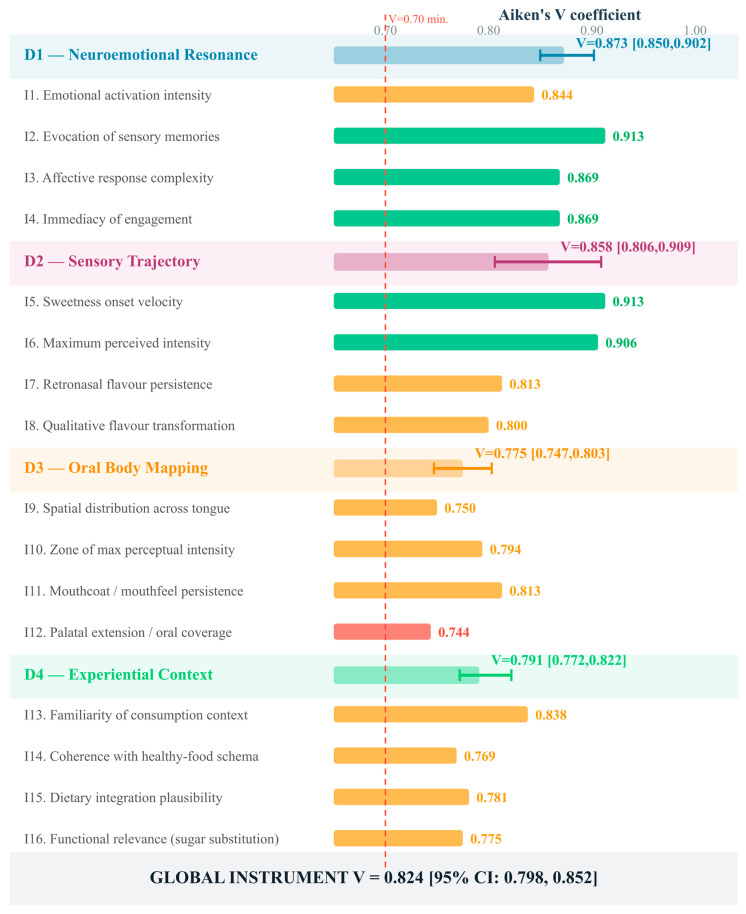
Content validity of the sensory instrument via Aiken’s *V* coefficient. Bars represent the *V*-value for each item across four perceptual dimensions: (D1) Neuro-emotional Resonance, (D2) Sensory Trajectory, (D3) Oral Body-Mapping, and (D4) Experiential Context. Error bars denote the 95% confidence intervals (*n* = 10 expert judges). The dashed red line indicates the minimum psychometric acceptability threshold (*V* = 0.70).

Content validity was established prior to data collection using Aiken’s *V* coefficient via a panel of 10 independent expert judges. The panel comprised university researchers and faculty members with graduate-level training in food science, sensory evaluation, consumer studies, and related disciplines, each possessing at least five years of academic, research, or professional experience in their respective areas of expertise. Experts were selected based on their demonstrated experience in sensory analysis or food-related research and independently evaluated the instrument. Ratings for Clarity, Pertinence, Relevance, and Sufficiency were collected on a 5-point Likert scale (Figure 1).

All 16 items individually attained *V* ≥ 0.744, exceeding the prospective threshold of *V* = 0.70 recommended for analytical sensory instruments. The global Aiken’s *V* was 0.824 (95% CI: [0.798; 0.852]; bootstrap *n* = 10,000 resamples, seed = 42), indicating robust expert consensus on construct alignment across the four validity criteria. Dimension-level *V* values ranged from *V* = 0.775 (D3—Oral Body-Mapping; 95% CI: [0.747; 0.803]) to *V* = 0.873 (D1—Neuro-emotional Resonance; 95% CI: [0.850; 0.902]).

The comparatively lower *V* of D3 reflects the inherent abstraction of introspective oral body-mapping instructions for evaluators unaccustomed to somatic spatial description; nonetheless, *V* = 0.775 exceeds the *V* ≥ 0.75 threshold recommended for analytical sensory constructs [23]. Within D2 (Sensory Trajectory), the two lowest individual *V* values correspond to items requiring temporally extended perceptual integration: I7 (retronasal flavor persistence; *V* = 0.813) and I8 (qualitative flavor transformation; *V* = 0.800), consistent with the documented cognitive complexity of retronasal olfaction under continuous evaluation paradigms.

### 2.3. Sensory Evaluation Environment and Conditions

Sensory assessments were conducted in a standardized sensory evaluation laboratory designed in strict accordance with ISO 8589:2010 [27]. Testing was performed within individual climate-controlled booths maintained at a constant temperature of 22 ± 1 °C and relative humidity of 55 ± 5%. Booths were equipped with artificial daylight-simulating illumination (6500 K) to neutralize visual bias, and the testing area was maintained under positive pressure with activated carbon filtration to guarantee an odor-free environment.

### 2.4. Sample Preparation, Presentation, and Scale Architecture

The enzymatically hydrolyzed yacon syrup was evaluated at its native production concentration of 15.3 °Brix (pH 4.7, reducing sugars 46.25 g/L), without external dilution, to capture its authentic rheological and organoleptic profile. Aliquots of 15 mL were served at a controlled temperature of 20 ± 1 °C in food-grade, transparent, odorless polypropylene cups. Each sample container was labeled with a unique, computer-generated three-digit random code to blind the assessors. Sample presentation followed a completely randomized, balanced monadic design to mitigate first-order and carryover positioning effects. The evaluation instrument consisted of a 16-item psychometric tool scored on a continuous 10.0 cm unstructured line scale. The left anchor corresponded to ‘Absent/Extremely Low’ (0.0 cm) and the right anchor corresponded to ‘Extremely Intense’ (10.0 cm), converted into quantitative scores with one decimal precision.

### 2.5. Panel Training and Calibration: Selection and Technical Qualification

The sensory panel was constituted through a rigorous two-stage sequential protocol aligned with ISO 11132:2021 [28] performance monitoring. From an initial pool of 38 candidates with prior descriptive analysis experience, a certified panel of 12 was selected based on repeatability and consensus alignment. In stage 1 (Precision), candidates performed triplicate evaluations of a reference syrup and were filtered by intra-individual Coefficient of Variation threshold (CV < 30%). Stage 2 (Accuracy), the multi-attribute Euclidean distance (*d*) to the group consensus vector was calculated, excluding candidates exceeding the threshold of *d* > 0.970 (Table 1).

The two-stage technical qualification protocol reduced the initial candidate pool (*n* = 38) to a certified panel of 12 evaluators (retention rate: 31.6%). In Stage 1 (CV criterion), 23 candidates were excluded: four classified as ‘exclude’ (CV ≥ 30%); the remainder presented CV values in the 20–30% range combined with marginal consensus distances. The Euclidean distance, 3 additional candidates were eliminated for distances exceeding the threshold (*d* > 0.970). The final panel exhibited CV values between 9.12% and 23.19% (mean = 15.96%) and consensus Euclidean distances between 0.518 and 0.969, confirming adequate intra-individual repeatability and alignment with the group centroid for all selected evaluators.

Following this screening, the 12 selected assessors underwent a structured 24 h training and calibration program distributed over 8 weeks, following ISO 11132:2021 guidelines. Training focused on attribute definition alignment, reference anchor anchoring for the four target dimensions, and scale-use synchronization to minimize individual variance. Panel calibration and stability were confirmed via Friedman’s test (*p* > 0.05 across three replicates), indicating high statistical consensus. Between evaluations, assessors executed a mandatory, rigorous palate-cleansing sequence consisting of a 30 s rinse with warm distilled water (30 ± 1 °C), followed by consumption of unsalted cracker fragments (matzo matrix), and a final rinse with room-temperature purified water, separating successive evaluations by a 5 min clearing window to prevent taste adaptation and sensory fatigue. All evaluated samples were safe and fit for human consumption. The study protocol was approved by the Institutional Ethics Committee of Universidad Nacional del Santa (CEI-UNS; Certificate No. CEI-UNS-FI-0001-2026; Project code PIC. FF. II. 003-2025), which classified the study as minimal-risk observational research in accordance with the Declaration of Helsinki (Fortaleza revision, 2013).

### 2.6. Statistical Analysis

Dimensional composite scores were computed as the arithmetic mean of the four constituent items per panelist per session. Descriptive statistics (mean, SD, CV) were derived at the item and composite dimension levels. Distributional integrity was verified using Shapiro–Wilk (normality) and Levene (homoscedasticity) tests. Between-dimension differences were evaluated using one-way ANOVA (F-statistic) and the heteroscedasticity-robust Kruskal–Wallis test (*H*-statistic), with dual reporting justified by the Levene result. Perceptual stability across sessions was assessed using Friedman’s repeated-measures test per dimension across three sessions. Pearson correlations between dimensions were computed with two-tailed significance tests.

PCA was applied to the standardized (*z*-score) 12 × 4 matrix via Singular Value Decomposition (SVD); component retention followed Kaiser’s criterion (eigenvalue λ ≥ 1.0), confirmed by parallel analysis. Communalities were calculated as the sum of squared factor loadings across retained components. Ward’s HCA was applied to the same standardized matrix using squared Euclidean distances; cluster number optimality was evaluated using the Calinski–Harabász pseudo-*F* statistic for *k* = 2 to 4; the cophenetic correlation coefficient assessed dendrogram representativeness. All analyses were executed in R software (version 4.6.0; R Core Team, Vienna, Austria) using the RStudio integrated development environment (v2026.01.1+403; Posit Software, Boston, MA, USA). PCA and HCA were employed as exploratory multivariate tools to visualize relationships among sensory dimensions and assess overall perceptual patterns within the trained panel. Given the limited number of certified assessors (*n* = 12), these analyses were interpreted descriptively and not as population-level inferential models. Their purpose was to support sensory pattern exploration rather than establish generalized latent structures.

## 3. Results

### 3.1. Descriptive Sensory Profile of the Yacon-Derived Fructose Syrup

The qualified panel (*n* = 12) established the initial sensory baseline for the matrix, as detailed in Table 2. At the item level, mouthcoat/oral coating persistence (I11: 6.49 ± 2.67) registered the highest mean across the entire instrument, followed by sweetness onset speed (I5: 6.53 ± 2.53), identifying these as the primary discriminative markers of the yacon syrup. Conversely, retronasal flavor persistence (I7: 4.54 ± 2.90) exhibited the highest intra-item CV (64.0%), reflecting the documented inter-individual heterogeneity in retronasal sensitivity for fructooligomeric matrices.

### 3.2. Descriptive Profile and Between-Dimension Comparison Tests

Dimensional composite means (Table 3) followed the rank order: D3—Oral Body-Mapping (5.493 ± 0.563) > D2—Sensory Trajectory (5.378 ± 0.855) > D4—Experiential Context (5.273 ± 0.728) > D1—Neuro-emotional Resonance (4.998 ± 0.368). D1 registered the lowest inter-individual variability (CV = 7.4%), indicating that the syrup elicits a coherent and uniform functional-associative schema independent of individual perceptual style. D2 exhibited the highest composite CV (15.9%) and the greatest item-level heterogeneity (item CV range: 44.1–68.7%).

Shapiro–Wilk tests confirmed normal distributions for all four-dimensional composites (*W* ≥ 0.844; *p* ≥ 0.131). Levene’s test detected no significant heteroscedasticity between dimensions (*F* = 0.552; *p* = 0.650), validating the use of parametric ANOVA as the primary test. One-way ANOVA yielded *F* = 2.363 (*p* = 0.084); Kruskal–Wallis test yielded *H* = 5.754 (*p* = 0.124). Both tests concurrently indicate the absence of statistically significant between-dimension mean differences, confirming dimensional homogeneity. This result is analytically meaningful: no single perceptual domain disproportionately dominates the global organoleptic response, producing the balanced sensory profile characteristic of commercially viable sweetener matrices.

### 3.3. Principal Component Analysis

**Figure 2:**
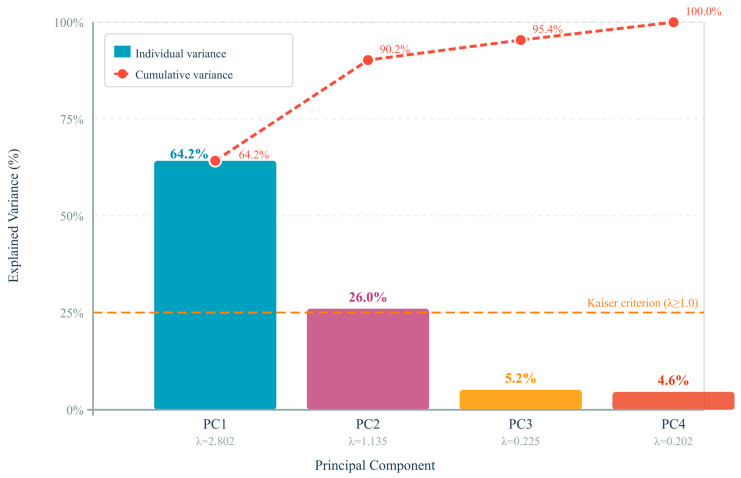
Scree plot of eigenvalues and variance distribution. The bar chart displays the individual variance explained by each principal component, while the dashed line tracks the cumulative explained variance. The retention of the first two components is supported by the Kaiser criterion (λ≥1), cumulatively accounting for 90.2% of the dataset’s variability.

The underlying dimensionality of the sensory dataset was determined through Principal Component Analysis (PCA). As illustrated in Figure 2, the Scree plot reveals a distinct elbow at the second component, with both PC1 and PC2 exceeding the Kaiser criterion (eigenvalue λ > 1). This two-component structure effectively captures 90.2% of the total variance, ensuring that the subsequent factor loadings and inter-dimensional correlations provide a high-fidelity representation of the syrup’s sensory space.

PCA applied to the standardized 12 × 4 dimensional composite matrix yielded a two-component solution satisfying Kaiser’s criterion (Table 4).

PC1 (*λ* = 2.802; 64.15% of total variance) received substantial positive factor loadings from D1 (0.560), D2 (0.574), and D3 (0.556), with a secondary contribution from D4 (0.220). This component is interpreted as a global somatic-integration axis: panelists with high PC1 scores exhibit simultaneously elevated sensory engagement across the neuro-emotional, temporal, and textural-somatic channels. The convergent validity of this interpretation is corroborated by the significant inter-dimensional Pearson correlations: D1–D2 (*r* = 0.727; *p* = 0.007), D1–D3 (*r* = 0.786; *p* = 0.002), and D2–D3 (*r* = 0.699; *p* = 0.010).

PC2 (λ = 1.136; 26.02% of total variance) was dominated by D4 (factor loading = 0.904), with minor negative contributions from D3 (−0.278) and near-zero contributions from D1 (−0.269) and D2 (0.184). This component is interpreted as a contextual-cognitive differentiation axis: panelists with high PC2 scores assign disproportionate weight to cognitive-associative processing (D4) while attenuating somatic-textural integration (D3). The orthogonality between PC1 and PC2 confirms that somatic integration and contextual cognition operate as independent sensory-cognitive processing channels in the evaluation of yacon syrup, with direct implications for understanding multidimensional sensory-directed formulation adjustments.

Communalities ranged from *h*^2^ = 0.363 (D2) to *h*^2^ = 0.865 (D4), confirming that D4 is almost entirely captured by the two-component solution. Cumulative explained variance reached 90.17%, exceeding the ≥85% reference criterion for multivariate studies in food science.

**Figure 3:**
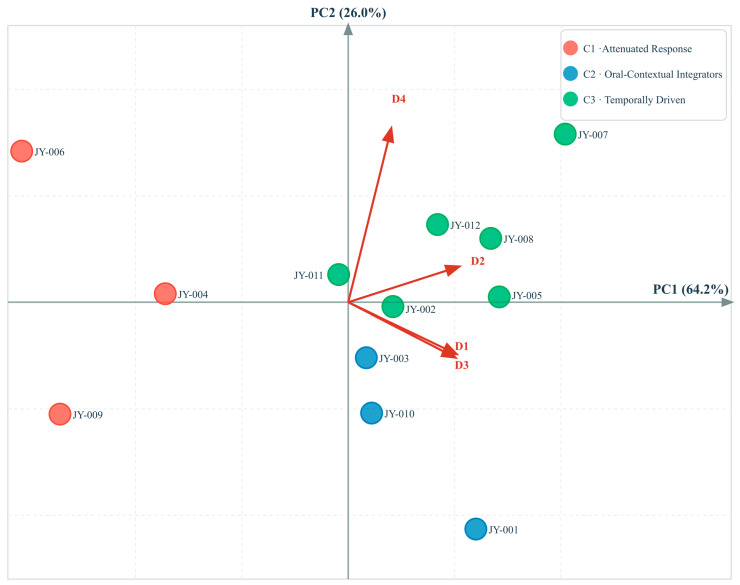
PCA Biplot—Sensory Space Consensus. The biplot illustrates the projection of the four sensory dimensions (vectors) and the individual scores of the 12 panelists (points) on the first two principal components. Panelists are color-coded according to their membership in the clusters identified via HCA: C1 (Attenuated Response), C2 (Oral-Contextual Integrators), and C3 (Temporally Driven).

The spatial distribution of the sensory dimensions and the individual panelist scores is visualized in the PCA Biplot (Figure 3). PC1, accounting for 64.2% of the variance, exhibits a strong convergence of the somatic and emotional vectors (D1, D2, and D3), while PC2 (26.0%) is almost exclusively defined by the experiential context (D4). This orthogonal arrangement confirms that the panelists’ cognitive perception regarding the experiential context (D4) operates independently from their immediate physical and temporal engagement during the tasting session.

**Figure 4:**
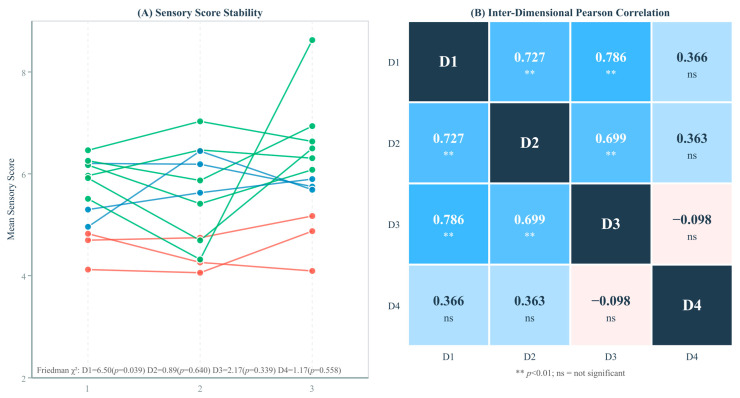
Panel reliability and inter-dimensional consistency metrics. (**A**) Sensory score stability across three experimental replicates, where colored lines trace the evaluation trajectories for each perceptual dimension (D1–D4); *p*-values from Friedman’s test indicate non-significant differences for dimensions D2, D3, and D4, confirming panel repeatability. (**B**) Pearson correlation heatmap between sensory dimensions; blue cells indicate significant positive associations (*p* < 0.01), pink cells denote negative associations, and pale cells denote non-significant correlations (ns), highlighting the orthogonality of the experiential context (D4) relative to the somatic-emotional axes.

The reliability of the sensory panel was assessed through a dual approach focusing on longitudinal stability and internal consistency. As shown in Figure 4, the longitudinal analysis (Panel A) revealed high perceptual stability across the three experimental replicates for the majority of the attributes, with no significant differences observed in dimensions D2, D3, and D4 (Friedman *p* > 0.05). This indicates a lack of fatigue or training decay during the sessions. Furthermore, the inter-dimensional correlation analysis (Panel B) identified strong, statistically significant associations between the somatic and temporal domains (D1, D2, and D3; *r* = 0.699), whereas D4 (Experiential context) remained statistically independent, confirming its role as a distinct cognitive axis in the sensory mapping of the matrix.

### 3.4. Hierarchical Cluster Analysis: Panelist Perceptual Segmentation

**Figure 5:**
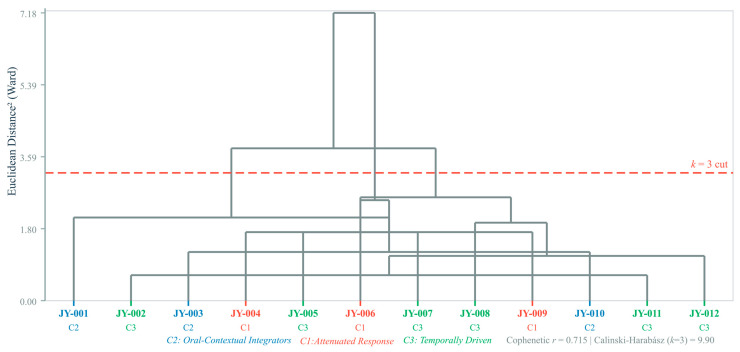
Ward’s HCA Dendrogram—Perceptual Cluster Segmentation. The dendrogram shows the hierarchical grouping of the 12 panelists based on their standardized sensory scores. The red dashed line indicates the optimal cut-off point (*k* = 3), identifying three well-separated clusters: C1 (Attenuated Response), C2 (Oral-Contextual Integrators), and C3 (Temporally Driven).

Ward’s Hierarchical Cluster Analysis (HCA) of the standardized 12 × 4 matrix was employed to identify distinct evaluator archetypes. As shown in Figure 5, the nodal structure and squared Euclidean distances support a three-cluster solution (*k* = 3), validated by a Calinski–Harabász pseudo-*F* index of 9.90 and a cophenetic correlation of *r* = 0.715. Although the *k* = 2 solution yielded a marginally higher pseudo-*F* (11.59), the *k* = 3 partition was deliberately selected based on the dendrogram’s clear nodal topology and its superior analytical interpretability for segment-specific formulation tracking. The classification information is visually derived from the vertical axis, which measures the dissimilarity between clusters; the red dashed line indicates the phenomenological cut-off point where between-cluster variance is optimized relative to within-cluster variance. Cluster profiles are synthesized in Table 5.

Cluster C1 (Attenuated Response; *n* = 3: JY-004, JY-006, JY-009) exhibited globally attenuated dimensional scores (D1 = 3.907; D2 = 5.140; D3 = 3.717; D4 = 5.389), positioned in the PC1-negative region of the biplot (Figure 1). The mean global score of C1 panelists (4.39) does not indicate active aversion, but insufficient perceptual engagement to activate pronounced intensity scoring—an analytically critical distinction for reformulation decisions, as this segment is unlikely to respond to incremental sensory optimization.

Cluster C2 (Oral-Contextual Integrators; *n* = 3: JY-001, JY-003, JY-010) was characterized by elevated scores in D3 (6.100) and D2 (6.513), with moderate D4 (4.842), positioned in the PC1-positive region of the biplot. These panelists represent an evaluator archetype in which oral textural completeness (mouthfeel dimensionality) and the temporal sweetness trajectory constitute the primary quality signals—providing critical baseline criteria for the standardization of functional yacon-derived sweetening systems.

Cluster C3 (Temporal Dominance; *n* = 6: JY-002, JY-005, JY-007, JY-008, JY-011, JY-012) showed the highest scores in D2 (6.654; sweetness onset speed I5 particularly elevated: 6.53) and D4 (6.386), consistent with a temporally sensitive and contextually coherent perceptual style. C3 is the numerically dominant archetype in the panel (50%), indicating it represents the primary sensory baseline for characterizing the multi-attribute profile of this formulation.

**Figure 6:**
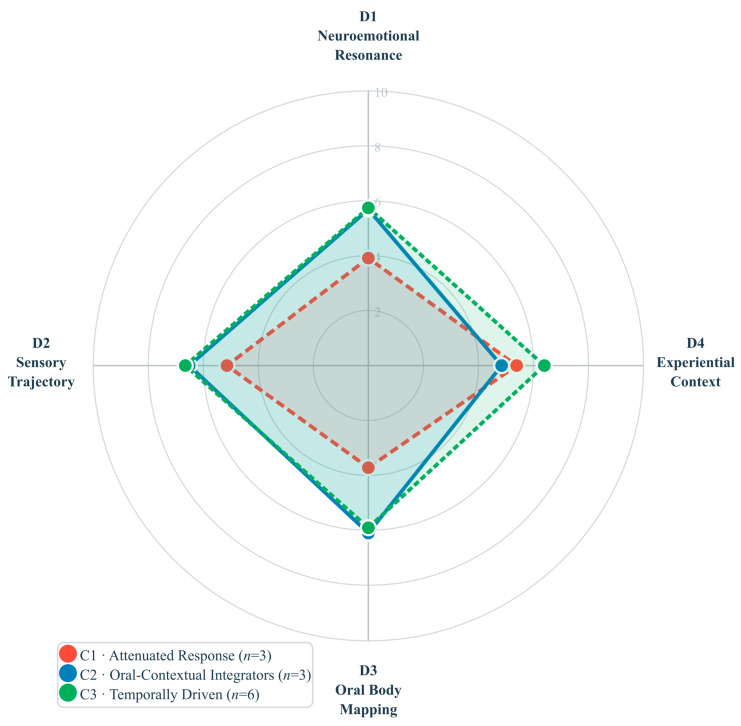
Multidimensional sensory fingerprints of the identified clusters. The radar chart illustrates the transition from a globally attenuated profile (C1) to a high-engagement archetype (C3), characterized by superior temporal sweetness kinetics and contextual coherence. This visualization confirms that the sensory acceptance of yacon syrup is driven by a balanced integration of oral body-mapping and experiential familiarity, providing a strategic map for targeted functional food positioning.

The divergent sensory profiles of the identified clusters are synthesized in the Perceptual Radar Chart (Figure 6). This multivariate visualization highlights the ‘multivariate profile’ of each segment, showing that while Cluster C3 (Temporally Driven) exhibits the highest engagement across all dimensions—particularly in sweetness kinetics—Cluster C1 remains globally attenuated. These distinct patterns confirm that panelist evaluation patterns for yacon syrup is not monolithic but is instead moderated by specific somatic and contextual sensitivities.

## 4. Discussion

### 4.1. Psychometric Qualification of the Sensory Panel and Construct Validity of the Instrument

The reduction in the panel from 38 to 12 evaluators, with a retention rate of 31.6%, reflects a rigorous selection process that strengthens the interpretative validity of the sensory results. The observed coefficients of variation between 9.12% and 23.19% (mean 15.96%) and Euclidean distances below 0.969 indicate adequate repeatability and strong alignment with the consensus centroid, minimizing random variability. This is particularly relevant in yacon matrices, where unfamiliar sensory characteristics may distort evaluations if panel calibration is insufficient. Previous studies have highlighted similar challenges in yacon-based products, where variability is linked to complex interactions between sweetness, texture, and functional perception [6,25]. Thus, the methodological rigor applied ensures that the variability observed reflects intrinsic product properties rather than evaluator inconsistency.

Regarding construct validity, the instrument demonstrated strong psychometric performance, with all items exceeding *V* ≥ 0.744 and a global Aiken’s *V* of 0.824, confirming coherence between descriptors and the evaluated construct. The slightly lower value in Oral Body-Mapping (*V* = 0.775) likely reflects the cognitive complexity of describing spatial oral sensations rather than a structural limitation. This interpretation is consistent with studies indicating that sensory descriptors involving introspection tend to show lower agreement due to abstraction demands [25]. Furthermore, high mean values in sweetness onset (I5 = 6.53) and mouthcoat persistence (I11 = 6.49) suggest that the instrument effectively captures both temporal and textural attributes, aligning with multidimensional approaches recommended for yacon-derived foods [3,14].

### 4.2. Multidimensional Sensory Balance and Temporal Dynamics

The absence of statistically significant differences between dimensions (ANOVA *p* = 0.084; Kruskal–Wallis *p* = 0.124) indicates that the syrup presents a statistical score homogeneity across the assessed perceptual domains under the tested conditions, rather than constituting definitive proof of an active functional equilibrium. Although sensory trajectory reached the highest mean (5.378 ± 0.855), the proximity to the remaining dimensions suggests an integrated response. This equilibrium is consistent with findings in yacon-enriched formulations, where sweetness interacts with texture and contextual associations rather than acting independently [28,29]. Additionally, the low variability observed in experiential context (CV = 13.8%) indicates that the product consistently activates a shared cognitive tracking pattern regarding the multi-attribute framework.

The prominence of sweetness onset (I5 = 6.53 ± 2.53) highlights the importance of early temporal perception in shaping the overall sensory experience. This behavior can be attributed to the presence of free fructose generated during partial hydrolysis of fructooligosaccharides, which contributes to rapid sweetness perception while oligomers modulate persistence [3,14]. The moderate value observed for retronasal persistence (I7 = 4.54 ± 2.90), together with its high variability (CV = 64.0%), suggests heterogeneous sensitivity among evaluators, a phenomenon previously reported in yacon-based matrices [25]. These results support the interpretation of a clean temporal profile, which has been associated with optimized discriminative attributes in functional sweeteners [29].

### 4.3. Perceptual Stability and Neuro-Emotional Variability

The stability observed in sensory trajectory, oral body-mapping, and experiential context across sessions (*p* > 0.05) indicates that the primary sensory attributes of the syrup are reproducible and resistant to fatigue or carry-over effects. This consistency reinforces the robustness of the panel and supports the reliability of the sensory profile obtained. Similar findings have been reported in yacon-based products, where sweetness perception and texture remain stable across repeated evaluations, suggesting that these attributes are structurally embedded in the product matrix [6,29]. Consequently, the stability of these dimensions strengthens their relevance for product characterization and optimization.

In contrast, Neuro-emotional Resonance exhibited significant variability (*p* = 0.039), accompanied by high coefficients of variation at the item level, reaching up to 68.7%. This indicates that emotional responses are more sensitive to contextual and individual factors than to intrinsic product properties. Previous research has shown that associative responses in yacon-derived foods is influenced by familiarity and perceived health benefits rather than purely sensory input [25,30]. Therefore, the observed variability should be interpreted as an inherent characteristic of emotional processing, which introduces flexibility in panelist evaluation patterns rather than undermining the validity of the results.

### 4.4. Multivariate Structure and Sensory-Cognitive Integration

The PCA results, explaining 90.17% of the total variance, reveal a highly structured perceptual system composed of two independent axes. The first component (64.15%) integrates neuro-emotional resonance, sensory trajectory, and oral body-mapping, supported by strong correlations (*r* up to 0.786), indicating that these dimensions operate as a unified sensory experience. This integration reflects the physicochemical behavior of yacon syrup, where sugars and fructooligosaccharides simultaneously influence taste and texture perception [3,14]. Similar integrative patterns have been reported in complex yacon matrices, where sensory perception emerges from interactions between compositional and structural factors [25].

The second component (26.02%), dominated by experiential context (loading 0.904; *h*^2^ = 0.865), defines a distinct cognitive axis associated with functional interpretation. Its orthogonality with the first component indicates that contextual evaluation operates independently from direct sensory stimulation. This separation is consistent with evidence showing that the multi-attribute profiling of yacon-based products depends not only on sensory quality but also on perceived physiological benefits, such as glycemic control and prebiotic effects [1,23]. Thus, the multivariate structure suggests that panelist perceptual mapping is shaped by both immediate sensory integration and higher-level cognitive evaluation.

### 4.5. Perceptual Segmentation and Evaluator Archetypes Derived from Hierarchical Cluster Analysis

The hierarchical cluster analysis revealed three distinct perceptual segments, confirming that sensory response to yacon syrup is not homogeneous but structured into differentiated evaluator profiles. Cluster C1 (Attenuated response) exhibited globally low scores across all dimensions (mean = 4.39), indicating limited perceptual engagement rather than explicit attribute discrimination. This pattern suggests that, for a subset of panelists, the syrup does not generate sufficiently strong sensory cues to trigger pronounced scoring criteria. Similar attenuation effects have been observed in products formulated with alternative sweeteners, where moderate sweetness and unfamiliar texture fail to activate clear discriminative sensory engagement [28,29]. From a formulation perspective, this segment represents a low-return target for sensory optimization alone.

Cluster C2 (Oral-contextual integrators) was characterized by elevated scores in oral body-mapping (D3 = 6.100) and sensory trajectory (D2 = 6.513), indicating a perceptual style strongly driven by mouthfeel completeness and sweetness evolution. This profile aligns with the known functional behavior of fructooligosaccharides, which contribute both to viscosity and sustained flavor perception in yacon-based systems [3,14]. Evaluators within this segment are likely to track textural richness and balanced sweetness tracking metrics, providing critical baseline criteria for the standardization of high-purity functional sweetening systems. Evidence from yacon-enriched formulations supports the importance of these attributes in driving multi-attribute tracking accuracy among calibrated assessors [4,30].

Cluster C3 (Temporal dominance), the largest group (50%), displayed the highest scores in sensory trajectory (D2 = 6.654) and experiential context (D4 = 6.386), reflecting sensitivity to sweetness onset and strong alignment with functional evaluation framework schemas. This indicates that rapid sweetness perception combined with clear health-related associations constitutes the primary descriptive tracking driver for the majority of evaluators. Previous studies have shown that yacon-derived products benefit from this dual perception, where immediate palatability reinforces the credibility of functional claims such as glycemic regulation and prebiotic activity [1,23]. Consequently, C3 representative sensory baseline for characterizing the multi-attribute profile of this formulation, as it integrates both sensory performance and cognitive validation, key factors for sensory standardization.

### 4.6. Segment-Oriented Formulation Strategies and Functional Optimization Pathways

The identification of three perceptual clusters enables the definition of targeted formulation strategies grounded in chemometric evidence rather than generalized optimization. For Cluster C2 (Oral-contextual integrators), the elevated scores in mouthfeel (D3) and temporal perception (D2) indicate that the current formulation—characterized by partial inulin hydrolysis and intermediate soluble solids (15.3 °Brix)—is already well aligned with segment evaluation profiles. Therefore, optimization efforts should focus on preserving the structural integrity of short-chain fructooligosaccharides (DP ≥ 3), which are responsible for viscosity and oral coating effects. Literature on yacon-derived syrups and concentrates confirms that maintaining this fraction is critical for both textural performance and prebiotic functionality, particularly under storage and processing conditions [3,4,14].

For Cluster C3 (Temporal dominance), which represents the largest proportion of evaluators, the key driver is sweetness kinetics rather than mouthfeel intensity. In this case, increasing the degree of enzymatic hydrolysis to enhance free fructose content could accelerate sweetness onset (I5) without fully eliminating the oligomeric fraction. This balance is essential, as excessive hydrolysis would reduce the functional and structural contributions of FOS. Studies on yacon-based beverages and reformulated foods indicate that optimizing the ratio between free sugars and oligomers improves both palatability and perceived naturalness, reinforcing panel tracking consistency in functional contexts [6,31]. Thus, process parameters such as hydrolysis time and enzyme activity become critical control points.

In contrast, Cluster C1 (Attenuated response) presents limited sensitivity to sensory improvements, suggesting that reformulation alone is unlikely to significantly increase intensity tracking engagement. Instead, this segment may respond more effectively to extrinsic product attributes, including nutritional labeling, health claims, and price positioning. Evidence from clinical and nutritional studies highlights the relevance of yacon in glycemic control, modulation of gut microbiota, and anti-inflammatory effects, which can be leveraged to enhance perceived value [1,23]. Consequently, a dual strategy emerges physicochemical optimization for high-response segments (C2 and C3), and concept-driven tracking for low-engagement consumers. This segmentation-based approach increases the efficiency of product development and sensory standardization.

## 5. Limitations

The present study is constrained by the limited panel size (*n* = 12), which places the multivariate analyses at the lower boundary of statistical robustness for sensory research. Although PCA explained 90.17% of the variance and HCA identified three clusters, these results should be interpreted cautiously, as small samples are sensitive to individual variability and cluster reassignment. The cophenetic correlation (*r* = 0.716) suggests an acceptable but not optimal representation of the distance matrix, indicating that future studies should incorporate larger panels and complementary techniques such as non-metric multidimensional scaling to improve structural validation. Additionally, the marginal significance observed in Neuro-emotional Resonance (*p* = 0.039) suggests that affective responses may require further methodological stabilization, potentially through the inclusion of anchoring stimuli to reduce variability across sessions.

Another limitation lies in the restricted scope of product evaluation, as only a single syrup formulation (15.3 °Brix; pH 4.7; fructose 35.22 g/L) was analyzed under fixed processing conditions. This prevents establishing how variations in hydrolysis degree, enzyme activity, or concentration influence the observed sensory dimensions, particularly the mouthcoat–retronasal asymmetry identified in the results. Moreover, the absence of direct chromatographic profiling of fructooligosaccharides limits the mechanistic interpretation of sensory attributes. Although the presence of short-chain FOS was inferred from processing parameters, techniques such as HPLC or mass spectrometry would be required to confirm chain-length distribution and its relationship with texture and persistence, as recommended in compositional studies of yacon matrices [12,13]. Finally, external validity is limited by both geographic sourcing and panel composition. The yacon tubers were obtained from a single high-altitude production zone (~3200 m a.s.l.), and previous research has shown that altitude, soil conditions, and harvest timing can significantly influence FOS composition and sugar profile [12,13]. Therefore, extrapolation of the sensory results to other origins should be approached with caution. In addition, the panel consisted of young, trained individuals from an academic environment, resulting in a homogeneous perceptual baseline. Consequently, the identified clusters (C1–C3) should be considered exploratory. Future studies should validate these findings using larger-scale affective testing with broader evaluator groups (*n* ≥ 100), incorporating tools such as Just-About-Right scales and penalty analysis to strengthen the formulation optimization framework.

## 6. Conclusions

This study established the multivariate sensory characterization and preliminary panel acceptability of an enzymatically hydrolyzed *Smallanthus sonchifolius* fructose syrup through a technically certified 12-member panel and a 16-item psychometric instrument with confirmed content validity (global Aiken’s *V* = 0.824; 95% CI [0.798; 0.852]). The empirical panel-derived data validated our central hypotheses, demonstrating that the targeted enzymatic conversion of yacon fructooligosaccharides generates a stable, defined sensory space characterized by repeatable descriptive dimensions across assessments.

One-way ANOVA and heteroscedasticity-robust Kruskal–Wallis tests confirmed statistical equivalence among the mean composite scores of the four perceptual domains. This lack of significance demonstrates score homogeneity under the tested conditions, rather than constituting definitive proof of an active functional equilibrium or balanced dominance across dimensions. Principal Component Analysis (PCA) successfully resolved 90.17% of total sensory variance into two orthogonal components: a global somatic-integration axis (PC1: 64.15%) and a contextual-cognitive differentiation axis (PC2: 26.02%). Hierarchical Cluster Analysis (HCA) segmented the panel into three distinct, reproducible structural sub-segments of hedonic behavior (*k* = 3; pseudo-*F* = 9.90; cophenetic *r* = 0.715).

Friedman’s repeated-measures test confirmed perceptual stability across three independent sessions (*p* ≥ 0.339), demonstrating high psychometric reliability and eliminating sensory fatigue as a confounding variable. Mouthcoat persistence (I11: 6.49 ± 2.67) and the mouthcoat–retronasal asymmetry ratio (1.43) were isolated as distinct structural sensory attributes of the syrup. These chemometric structures confirm that the exploratory formulation exhibits consistent sensory attributes that positively influence the trained panel’s overall multidimensional sensory profile, providing a verified, reproducible baseline for the sensory standardization of functional yacon-derived products. Future work involving systematic chromatographic profiling is required to formally correlate specific fructooligosaccharide chain-length distributions with individual kinetic sweetness and mouthfeel parameters.

## Figures and Tables

**Table 1:** Panel constitution and selection metrics.

Panelist	Mean	SD	CV (%)	Euclidean Distance	Technical Criterion
SEA-005	5.714	0.717	12.55	0.718	Strong candidate → final panelist
SEA-009	5.095	0.768	15.08	0.621	Strong candidate → final panelist
SEA-011	4.905	0.768	15.67	0.518	Strong candidate → final panelist
SEA-013	5.048	0.805	15.94	0.648	Strong candidate → final panelist
SEA-014	4.667	0.796	17.05	0.714	Strong candidate → final panelist
SEA-023	5.571	1.076	19.31	0.969	Strong candidate → final panelist
SEA-016	4.810	0.981	20.39	0.774	Acceptable candidate → final panelist
SEA-020	4.857	1.108	22.82	0.772	Acceptable candidate → final panelist
SEA-012	4.619	1.071	23.19	0.886	Acceptable candidate → final panelist
SEB-006	6.333	0.577	9.12	0.800	Strong candidate → final panelist
SEB-015	6.238	0.625	10.02	0.794	Strong candidate → final panelist
SEB-014	5.810	0.602	10.36	0.613	Strong candidate → final panelist

Note: SEA, SEB = code of participant; SD = standard deviation; CV = coefficient of variation (SD/mean × 100%). ISO 11132:2021 thresholds: CV < 20% = strong candidate; 20% ≤ CV < 30% = acceptable; CV ≥ 30% = excluded. Euclidean distance to the group consensus centroid was the Stage 2 exclusion criterion (*d* ≤ 0.969).

**Table 2:** Sensory instrument structure, item-level descriptive statistics (*n* = 12 panelists).

Dim.	Item	Sensory Descriptor	Mean	SD	CV (%)
D1	I1	Emotional activation intensity	5.77	3.29	57.1
	I2	Evocation of prior sensory memories	5.06	2.23	44.1
	I3	Complexity of affective response	4.58	2.96	64.6
	I4	Immediacy of emotional engagement	4.58	3.15	68.7
D2	I5	Sweetness onset speed	6.53	2.53	38.7
	I6	Maximum perceived intensity $(T_{max}$)	4.98	2.86	57.5
	I7	Retronasal flavor persistence (aftertaste)	4.54	2.90	64.0
	I8	Qualitative flavor transformation	5.46	3.36	61.5
D3	I9	Spatial distribution on the tongue	4.93	2.72	55.2
	I10	Zone of maximum perceptual intensity	4.96	3.22	64.9
	I11	Mouthcoat/oral coating persistence	6.49	2.67	41.1
	I12	Palatal extension (oral coverage)	4.71	2.19	46.5
D4	I13	Familiarity of consumption context	5.58	2.63	47.1
	I14	Coherence with the healthy-food schema	5.92	2.60	43.9
	I15	Plausibility of dietary integration	5.44	2.88	53.0
	I16	Functional relevance (sugar substitution)	5.03	3.04	60.4

Note: SD = standard deviation; CV = coefficient of variation.

**Table 3:** Distributional assumption tests and between-dimension comparison for dimensional composite scores.

Composite Dimension	Mean	SD	CV (%)	W (S–W)	*p* (S–W)	Distribution	Homoscedasticity
D1: Neuro-emotional Resonance	4.998	0.368	7.4	0.844	0.131	Normal	Levene F = 0.552
D2: Sensory Trajectory	5.378	0.855	15.9	0.913	0.983	Normal	*p* = 0.650
D3: Oral Body-Mapping	5.493	0.563	10.2	0.869	0.555	Normal	(homoscedastic)
D4: Experiential Context	5.273	0.728	13.8	0.869	0.669	Normal	—

Note: S–W = Shapiro–Wilk *W* test statistic; Levene *F* = Levene’s test statistic; *p* > 0.05 indicates that normality and= homoscedasticity assumptions are statistically retained.

**Table 4:** PCA factor loadings, eigenvalues, communalities, and inter-dimensional Pearson correlations.

Composite Dimension	PC1	PC2	PC3	PC4	*h* ^2^	Pearson Correlations (r)
D1: Neuro-emotional Resonance	0.560	−0.269	−0.499	−0.604	0.386	D1–D2: *r* = 0.727 **; D1–D3: *r* = 0.786 **
D2: Sensory Trajectory	0.574	0.184	−0.333	0.725	0.363	D2–D3: *r* = 0.699 **; D2–D4: *r* = 0.363 ns
D3: Oral Body-Mapping	0.556	−0.278	0.784	−0.009	0.386	D1–D4: *r* = 0.366 ns
D4: Experiential Context	0.220	0.904	0.160	−0.330	0.865	D3–D4: *r* = −0.098 ns
Eigenvalue (λ)	2.802	1.135	0.225	0.202	—	—
Variance (%)	64.15	26.02	5.21	4.62	—	—
Cumulative variance (%)	64.15	90.17	95.38	100	—	—

Note. Extraction via SVD (Singular Value Decomposition) on standardized composites (*z*-score; *n* = 12). Component retention: Kaiser’s criterion (λ ≥ 1.0) confirmed by parallel analysis. Loadings > |0.40| are primary. Communality *h*^2^ = sum of squared loadings on PC1 and PC2. ** *p* < 0.01; ns = not significant (α = 0.05). Cumulative variance PC1 + PC2 = 90.17%, exceeding the ≥85% reference threshold.

**Table 5:** Cluster profiles from Ward’s HCA (squared Euclidean distances; *k* = 3; *n* = 12 panelists).

Cluster	*n*	Members	D1	D2	D3	D4	Perceptual Profile
C1: Attenuated Response	3	JY-004, JY-006, JY-009	3.907	5.140	3.717	5.389	Globally conservative intensity scoring; uniform attribute tracking without extreme valuations.
C2: Oral-Contextual Integrators	3	JY-001, JY-003, JY-010	5.681	6.513 ✦	6.100 ✦	4.842	High mouthcoat + temporal trajectory; functional food evaluator archetype.
C3: Temporal Dominance	6	JY-002, JY-005, JY-007, JY-008, JY-011, JY-012	5.741	6.654 ✦	5.910	6.386 ✦	Prominent sweetness kinetics + contextual coherence; dominant analytical profile within (50%).

Note. D1–D4 = arithmetic means of dimensional composite scores for panelists within each cluster. ✦ = highest-scoring dimension within the cluster. Calinski–Harabász pseudo-*F*: *k* = 2 → 11.59; *k* = 3 → 9.90; *k* = 4 → 8.29. Cophenetic correlation coefficient *r* = 0.715.

## Data Availability

The data presented in this study are available on request from the corresponding author due to privacy or ethical restrictions.

## Abbreviations

The following abbreviations are used in this manuscript:

FOS	Fructooligosaccharides
DP	Degree of polymerization
PCA	Principal Component Analysis
HCA	Hierarchical Cluster Analysis
SVD	Singular Value Decomposition
PC	Principal component
CV	Coefficient of variation
SD	Standard deviation
ANOVA	Analysis of variance
JAR	Just-About-Right (hedonic scale)
PREFMAP	External preference mapping
NMDS	Non-metric multidimensional scaling
HPLC	High-performance liquid chromatography
HFCS	High-fructose corn syrup
ISO	International Organization for Standardization
